# Intraoperative ultrasound in breast cancer surgery—from localization of non-palpable tumors to objectively measurable excision

**DOI:** 10.1186/s12957-018-1488-1

**Published:** 2018-09-11

**Authors:** Natasa Colakovic, Darko Zdravkovic, Zlatko Skuric, Davor Mrda, Jasna Gacic, Nebojsa Ivanovic

**Affiliations:** 1 Department of Surgical Oncology, University Medical Center “Bezanijska Kosa”, Bezanijska kosa bb, Belgrade, 11080 Serbia; 2 Faculty of Medicine, University of Belgrade, Belgrade, Serbia; 3 Department of Radiology, University Medical Center “Bezanijska Kosa”, Belgrade, Serbia

**Keywords:** Breast cancer excision, Intraoperative ultrasound, Tumor localization

## Abstract

**Background:**

The utilization of intraoperative ultrasound (IOUS) in breast cancer surgery is a relatively new concept in surgical oncology. Over the last few decades, the field of breast cancer surgery has been striving for a more rational approach, directing its efforts towards removing the tumor entirely yet sparing tissue and structures not infiltrated by tumor cells. Further progress in objectivity and optimization of breast cancer excision is possible if we make the tumor and surrounding tissue visible and measurable in real time, during the course of the operation; IOUS seems to be the optimal solution to this complex requirement. IOUS was introduced into clinical practice as a device for visualization of non-palpable tumors, and compared to wire-guided localization (WGL), IOUS was always at least a viable, or much better alternative, in terms of both precision in identification and resection and for patients’ and surgeons’ comfort. In recent years, intraoperative ultrasound has been used in the surgery of palpable tumors to optimize resection procedures and overcome the disadvantages of classic palpation guided surgery.

**Objective:**

The aim of this review is to show the role of IOUS in contemporary breast cancer surgery and its changes over time.

**Methods:**

A PubMed database comprehensive search was conducted to identify all relevant articles according to assigned key words.

**Conclusion:**

Over time, the use of IOUS has been transformed from being the means of localizing non-palpable lesions to an instrument yielding a reduced number of positive resection margins, with a smaller volume of healthy breast tissue excided around tumor, by making the excision of the tumor optimal and objectively measurable.

## Background

For a number of years, surgery has been essential in achieving a satisfactory level of locoregional control in patients with breast cancer, especially utilizing radical mastectomy and its modifications. In the last decades, the field of surgical oncology has been striving for a more rational approach, directing its efforts towards removing the tumor entirely, while sparing tissue and structures not infiltrated by tumor cells. This contributes to better functional and esthetic results and, by extension, a better quality of life for the patient.

Two main techniques that highlight the shift from classical to modern breast cancer surgery are breast-conserving surgery and sentinel node biopsy. The former benefits patients esthetically and psychologically, while the latter helps to improve functional results, primarily preventing limited motility and edema of the arm.

The institution of screening programs and the advancement of diagnostic methods significantly increased the percentage of tumors discovered in an early phase while still relatively small in size, effectively allowing for less radical procedures than the overly extensive “quadrantectomy.” Another consequence of discovering tumors in an early phase is an increase in the number of non-palpable lesions; the surgeon thereby needing additional tools for the localization and adequate excision of such lesions. Two main approaches to this are preoperative (wire- and radio-guided occult lesion localization by mammography: WGL and ROLL) and intraoperative (intraoperative ultrasound: IOUS).

For palpable lesions, localization is easily defined by palpation. However, adequate excision, negative margins with as small excision volume as is possible, may be better achieved if the distance from the tumor border to the resection margin is objectively measured by IOUS, rather than by subjective palpation-guided surgery.

It is clear that palpation-guided surgery cannot improve objectivity and measurability of the resection procedure. If we desire progress in objectivity and optimization of breast cancer excision, we need to make the tumor and the surround tissue visible and measurable during the course of the operation in real time. IOUS seems to be the optimal solution to this complex requirement.

In clinical practice, there are three main purposes of using IUOS in breast surgery: (1) localization of non-palpable lesions, (2) achieving free resection margins, and (3) IOUS-guided surgery in an attempt to obtain optimal excision volume, with negative resection margins and minimal sacrifice of surrounding healthy tissue.

The aim of this review is to highlight the role of IOUS in contemporary breast cancer surgery and its changes over time, from being a tool for the localization of non-palpable lesions, to becoming an instrument that can make tumor excision optimal and even objectively measurable.

## Main text

### IOUS in localization of non-palpable tumors

The utilization of intraoperative ultrasound in breast cancer surgery is a relatively new concept in this field of surgical oncology. It was introduced into clinical practice as a device for the visualization of non-palpable tumors [1–11]. All studies published thus far, including newly published studies [12–15], have shown an efficacy of almost 100% (Table 1).

**Table 1:** Identification rate of a non-palpable breast lesion by IOUS and WGL

Author	Type of the study	IOUS				WGL			
		N^o^ of patients	N^o^ of operations	N^o^ of ident. tumors	Ident. rate (%)	N^o^ of patients	N^o^ of operations	N^o^ of ident. tumors	Ident. rate (%)
Rahusen	prosp	19	20	20	100	43	43	43	100
Snider	retro	22	22	22	100	22	22	22	100
Harlow	retro	62	65	65	100	nd	nd	nd	nd
Smith	retro	81	81	81	100	nd	nd	nd	nd
Rahusen 2	prosp	27	27	27	100	22	22	22	100
Kaufman	prosp	100	101	101	100	nd	nd	nd	nd
Gittleman	retro	15	15	15	100	nd	nd	nd	nd
Beneth	prosp	103	115	115	100	24	24	24	100
Haid	retro	299	299	299	100	61	61	61	100
Potter	retro	32	32	32	100	nd	nd	nd	nd
Ngo	prosp	70	70	67	96	nd	nd	nd	nd
Fortunato	prosp	77	77	77	100	nd	nd	nd	nd
James	retro	96	96	96	100	59	59	59	100
Bouton	retro	28	28	28	100	nd	nd	nd	nd
Berentz	prosp	120	120	120	100	138	138	138	100
Ramos	retro	225	225	224	99	nd	nd	nd	nd

*IOUS* Intraoperative ultrasound, *WGL* wire-guided localization, *nd* no data, *N*^*o*^ number, *ident* identified, *ident. rate* identification rate

The first report was published by Schwartz et al. [1] in 1988 on 92 excised non-palpable breast lesions. They concluded that IOUS had proven effective and accurate, and that in select patients, it may be used in addition to, or instead of, X-ray needle localization for the precise excision of non-palpable breast lesions, excluding calcifications.

The next reports dealing with IOUS appeared more than 10 years later, mostly comparing WGL and IOUS [2, 3, 5]. In two reports [2, 5] with a relatively small number of operated lesions (63 in first study and 49 in the second, the second being a randomized trial) Rahusen et al. showed remarkable difference between IOUS and WGL in the adequacy of resection margins. In the IOUS group, resection margins were negative in 89% in both studies, compared to only 40% and 55% in the WGL group. Snider and Morrison [3] presented their small study on 44 patients, 22 in both IOUS and WGL groups. Both groups had the same number of positive resection margins, but the mean resection volume was smaller in the IOUS group (62.6 versus 81.1 cm^3^), although mean lesion size was two times larger in the IOUS group than in the WGL group (11 versus 5.5 mm). Smith et al. [4] emphasized that using IOUS avoids the complications of WGL and simplifies the scheduling of surgical procedures. This is a common sentiment among all authors dealing with this topic.

In the following years, very few studies on using IOUS in breast lesion surgery were published. Kaufman et al. [6] reported a series of 101 operations of non-palpable carcinomas in which they had a 100% identification rate. Bennett et al. [7] published a study on 115 resected non-palpable breast lesions of which 42% were malignant. The identification rate for all lesions was 100%. Negative resection margins were achieved in 93% of 48 excised lesions. This was retrospectively compared with hookwire-guided excisions performed by the same author, where negative resection margins were achieved in 83% of cases out of 43 operated malignant lesions. Haid et al. [8] reported 100% efficacy in the identification of occult breast cancer in 299 patients, and the same efficacy was reached in a control WGL group of 61 patients. Potter et al. [9] had the same maximum rate for 32 patients. In a prospective study, Ngo et al. [10] reached a 95.7% identification rate for 70 patients with impalpable lesions. They missed tumors less than 5 mm in diameter in two patients with body mass indexes over 25. In a prospective study, Fortunato et al. [11] achieved a 100% identification rate for 77 patients (60 malignant and 17 benign). Ramos et al. [14] had a 99.6% identification rate in a retrospective study on 225 invasive breast cancers. Only one tumor smaller than 5 mm could not be located.

### IOUS and resection margins

The use of IOUS to guide surgical excision of non-palpable breast carcinoma has also shown that ultrasound-guided breast cancer operations yield a smaller number of positive resection margins [2, 4–8, 15–17]. They also result in a smaller volume of healthy breast tissue excised around the tumor (Table 2).

**Table 2:** Tumor-free resection margins and re-excision rate after IOUS and WGL

Author	Type of the Study	IOUS				WGL			
		N^o^ of pts.	N^o^ of oper	N^o^ of neg. marg.	N^o^ of re-excision	N^o^ of pts.	N^o^ of oper	N^o^ of neg. marg.	N^o^ of re-excision
Rahusen	prosp	19	20	17 (89%)	nd	43	43	17 (40%)	nd
Snider	retro	22	22	18 (82%)	nd	22	22	18 (82%)	nd
Harlow	retro	62	65	63 (97%)	3(4.80%)	nd	nd	nd	nd
Smith	retro	81	81	24/25 mg (96%)	nd	nd	nd	nd	nd
Rahusen 2	prosp	27	27	24 (89%)	nd	22	22	12(55%)	nd
Kaufman	prosp	100	101	90(89%)	9 (9%)	nd	nd	nd	nd
Gittleman	retro	15	15	14(92%)	1(8%)	nd	nd	nd	nd
Beneth	prosp	103	115	39/42 mg (93%)	3(7%)	24	24	19 (83%)	5(17%)
Haid	retro	299	299	242 (81%)	57(19%)	61	61	38 (62%)	23 38%)
Potter	retro	32	32	28(88%)	nd	nd	nd	nd	nd
Ngo	prosp	70	70	66 (94%)	3(4%)	nd	nd	nd	nd
Fortunato	prosp	77	77	75 (97%)	2(3%)	nd	nd	nd	nd
Bouton	retro	28	28	25 (91%)	3(9%)	nd	nd	nd	26%
Berentz	prosp	120	120	112 (93%)	15(13%)	138	138	129(93.5%)	15(11%)
Ramos	retro	225	225	216 (96%)	9(4%)	nd	nd	12(55%)	nd
James	retro	96	96	10(10%)	20(20%)	59	59	52(88%)	18(30%)

*N*^*o*^ Number, *IOUS* intraoperative ultrasound, *WGL* wire-guided localization, *nd* no data, *pts.* patients, *prosp* prospective, *retro* retrospective, *neg.marg.* negative margins, *oper* operations

In a study on 65 breast cancers by Harlow et al. [15], the authors reported only two positive margins with a mean distance of 0.8 cm to the closest margin of excision. Moore et al. [16] reported their prospective study evaluating surgical accuracy and margin status after lumpectomies for palpable breast cancer on two groups of patients. In one group, they used IOUS (*n* = 27) but not in the other (*n* = 24). In the first group, only one patient had a positive margin (1/27 or 3.7%); while in the other group, seven patients had positive margins (7/24 or 29%). The authors concluded that the use of ultrasound-guided surgery optimizes the surgeon’s ability to obtain satisfactory margins for breast-conserving techniques in patients with breast cancer and that patient satisfaction with the cosmetic results was excellent. In the Kaufman et al. [6] study, negative margins for invasive carcinoma were found in 90% of patients; while in the Bennett et al. [7] study, resection margins were adequate in 93% of operations for malignant tumors.

Most authors compared IOUS and WGL in achieving negative resection margins. Haid et al. [8] reported 81% successful operations in IOUS group without metachronous secondary surgery, versus 62% in a WGL group. James et al. [18], in the only study related exclusively to DCIS, reported non-significant differences in resection adequacy between IUOS (96 pts.—10.4% of positive margins) and WGL (59 pts.—11.9% of positive margins). In a retrospective analysis, Bouton et al. [19] found that 28 patients treated by WGL and IOUS (control group was treated by WGL only) had a lower rate of positive margins (9% vs. 26%). Davis et al. [20] in a retrospective study (22 pts. with IOUS and 44 pts. without; tumors were palpable) showed that the IOUS group had significantly less involved margins (9% vs. 41%) and a lower rate of re-excision (9% vs. 34%).

Two studies showed no differences between IOUS and other techniques. In a prospective study on non-palpable tumors, Berentsz et al. [12] compared IUOS and WGL. There were 120 pts. in the IOUS group, and 138 pts. in the WGL group. Tumor-free resection margins were obtained in 93.5% of cases in the WGL group and 93.3% in the IOUS group. It is surprising that in this study, the average diameter of impalpable tumors in the IOUS group was 1.24 cm. Similar results were reported by Fisher et al. [17] in a retrospective analysis comparing resection margins in 73 patients with palpable tumors operated by IOUS-guided surgery and 124 patients operated by palpation guided surgery. Re-excision rates were similar in both groups, 17 (23%) in the IOUS group versus 31 (25%) in the palpation group. Nevertheless, the authors concluded that US guidance provides an excellent tool to aid the breast surgeon.

Eichler et al. [21] had more R0 resections in the IOUS group (84 pts.) than in the control group (without IUOS group 166 pts.), a statistically significant difference. In a retrospective analysis by Yu et al. [13], positive margins were found in only 9.29% of 126 palpable and 255 non-palpable tumors operated by IOUS guidance. In another retrospective analysis of 225 operated non-palpable tumors, Ramos et al. [14] had a re-excision rate of only 4% (9/225) after IOUS-guided surgery.

### IOUS-guided surgery

Improved margin status after IOUS-guided surgery for non-palpable tumors have initiated the application of this technique in the surgery of palpable tumors in recent years [12, 13, 17, 20–24], in order to optimize resection procedures and overcome the disadvantages of classic palpation guided surgery. Palpation-guided surgery is a subjective technique, yielding up to 41% of “positive” resection margins according to Krekel et al. [24] while leading to an unnecessary large volume of excision.

All published studies on this topic have unequivocally shown that intraoperative ultrasound improves oncological efficacy and cosmetic outcomes in breast conserving surgery. Olsha et al. [22] concluded that intraoperative ultrasound may help maintain low incidence of reoperation after breast-conserving surgery. In a paper by Davis et al. [20], the authors found that patients who underwent lumpectomies using IOUS were less likely to have an involved margin or to require re-excision. The lumpectomy volumes in the IOUS group were smaller than in the lumpectomy alone group. IOUS can decrease the rate of positive margins and re-excision lumpectomy in patients with palpable breast cancers. Fisher et al. [17] stated that although palpable breast cancers can be excised based on direct palpation or needle localization, ultrasound guidance provides an excellent tool to aid the surgeon. Only 10% of patients in the ultrasound-guided group had a positive margin in final pathology compared to 16% in the palpation-guided group. The re-excision rates were similar for both groups, 23% in the ultrasound-guided group versus 25% in the palpation-guided group. However, the rate of residual disease in re-excision pathology for a positive or close margin was significantly lower for those patients who had an ultrasound-guided lumpectomy than for those who had a palpation-guided lumpectomy.

In the COBALT trial, Krekel et al. [24] showed that the intraoperative use of IOUS for palpable tumors is associated with a 15% reduction in “positive” margins of resection. It also significantly reduced specimen volume when compared to palpation-guided surgery, leading to a more acceptable esthetic result and better quality of life.

### Surgical techniques of IOUS guides surgery

The IOUS surgical technique described thus far normally relies on the surgeon to mark the projected tumor margins on the skin of the breast before the first incision is made. The surgeon then inserts the probe into the wound multiple times in an effort to determine the relation between the tumor and the surrounding tissue once surgery proper has started. Once the excision has been completed, the specimen is examined ex vivo (i.e., ultrasound examination of the excised specimen), followed by additional shaving excisions if one of the excision margins if found to be too close to the edge of the tumor. In the COBALT trial, the authors used ultrasound during the entire procedure in order to gauge the distance of the resection line from the edge of the tumor in all directions and the entirety of the volume of the specimen without using any marker inside or around tumor.

Some authors have proposed the use of markers as an anatomical landmark, without any desire for them to aid fine measurements and resection line planning. Kaufman et al. [6] used die and wire needles to mark the position of the tumor. Gittleman [25] described 15 resections in which he had injected the tumor with an ultrasound contrast medium, 9 resections in which he had utilized a radiofrequency localization device (comprising a calibrated shaft with a flexible cutting element to facilitate the positioning of the device and fixing wires that expand radially in order to anchor the device on the target lesion), and 6 cases in which he had opted for an 18G needle as a means of marking the position of the tumor.

Using intraoperative ultrasound in such a “standard” way has been demonstrated by Ivanovic et al. [26, 27] to be fraught with difficulties that interfere with the comfort and precision of the surgical procedure. First, marking the position of the tumor by projecting its margins on the skin is problematic at best, given that the anatomical relations between the tumor and the surrounding tissue changes due to tissue retraction and manipulation normally involved in any type of surgery. Second, once the surgeon starts resecting the tumor, air and fluid in the wound create artifacts that significantly reduce the quality of the ultrasound image, therefore limiting useful interpretation. Third, when the ultrasound probe is inserted into the wound, the surrounding tissue is displaced and compressed which may lead to the surgeon misjudging the distance from the resection line to the edge of the tumor. Fourth, ultrasound refraction which is particularly common when scanning tissue that is irregular in shape, such as a tumor, leads to a discrepancy between the ultrasound-measured size of the tumor and its real size. This is most common in tumors 2 cm or larger. Since documenting this phenomenon, De Jean et al. [28] recommend that an ultrasound contrast medium be inserted into the tumor in relation to which segmental measurements in all directions can be performed.

In the same paper [27], Ivanovic et al. presented an original technique for the optimization of breast cancer excision (both palpable and non-palpable) utilizing IOUS and a specially-constructed needle as a marker for objective measurement. Guided by ultrasound, the needle is inserted into the tumor (the patient lying on the operating table anesthetized) and then used to measure the distance between the line of resection and the needle in all directions. The surgeon then proceeds to do the resection using these measurements while continually measuring the distance of the resection line from the needle, using a sterile ruler.

The preliminary results are encouraging, and it seems that the utilization of the aforementioned technique makes the resection of a breast cancer a measurable and objective undertaking. This should lead to a reduction in the percent of “positive” resection margins, and by extension, relapses. Viewed from a different perspective, one could expect improved conservation of healthy tissue, which should lead to smaller tissue defect and better esthetic results of the surgery. The authors consider the technique to be simple, easy to learn and implement, and comfortable for the surgeon. There is no need to palpate or compress the tumor or the surrounding tissue, and the traction, manipulation, and separation of the tissue is gentler than with palpation-guided surgery. Probably, the only drawback is the extra time needed for measurements before the incision (11 min on average at the moment). However, this time is compensated by the ease with which the resection is done and by the fact that it is done in a more rational and objectively measurable way. One could expect that, with training, the time needed for measurements and the resection itself will be shortened and that the relations (between the desired and achieved size of the tumor specimen) will become more optimal. Nevertheless, we must conclude that conducting a randomized trial is the only way to prove these assumptions.

### Discussion

Intraoperative ultrasound in breast cancer is a relatively new technique in this field of surgical oncology. It was introduced into clinical practice as a device for the visualization and localization of non-palpable tumors, and its utility and accuracy for this has always been unequivocally confirmed [1–11]. IOUS-guided surgery improves the accuracy and quality of classical surgery, while at the same time being cheap, time-efficient, simple, and comfortable for both the surgeon and the patient. There is no risk of complications related to the procedure, and thanks to greater precision, it is less likely that subsequent operations will be required.

In addition to being a non-radioactive technique, real-time visualization overcomes the shortcomings of standard preoperative mammography in a number of ways. First, it solves an organizational and technical problem by harmonizing the work of the diagnostics and operating rooms. Second, it reduces pre-surgery psychological stress for the patient, as there is no need for a painful and harsh procedure of breast compression and puncture while conscious. Third, it resolves the inability to check marker position after placement. This is important as there may be a movement of needle marker on the way from the diagnostics room to the operating room, while preparing the operating area before surgery [27].

Data analysis in studies on the use of IOUS to guide surgical excision of non-palpable breast carcinoma has also shown that IOUS-guided breast cancer operations yield a smaller number of positive resection margins [2, 4–7, 15–17]. This effect was unintended and unexpected in some studies [17] but nevertheless pointed to another possibly useful role of IOUS.

Studies comparing IOUS and WGL in achieving negative resection margins [8, 12, 18–20] showed that IOUS is at least equal or more successful than WGL. However, WGL is still a standard approach in the localization of non-palpable breast lesions and is currently irreplaceable when these lesions are invisible to ultrasound. IUOS could be a much better alternative to WGL for ultrasound-visible breast lesions in terms of precision in identification and resection as well as the comfort of patients and surgeons. Knowing this, it is surprising that IOUS-guided surgery is not more commonly used in breast cancer surgery of non-palpable lesions and that WGL is still the method of choice for localization of these lesions. One possible reason could be the lack of surgeons’ education in the use of ultrasound [24], which indicates the possible need for workshops and other forms of continuing education. Also, it seems that creating guidelines for optimal tumor excision in breast cancer surgery persuade surgeons to use IOUS more in the future.

The next step in the evolving role of IOUS in breast cancer surgery is “optimal and objectively measurable tumor excision” to achieve two main goals: first, a negative resection margin; and second, minimal sacrifice of surrounding healthy tissue, which improves the esthetic effect of the operation and patients’ quality of life. This has been illustrated by contemporary studies where IUOS were used as a means of optimal resection [24, 27], defined as a macroscopic distance of 10 mm in all directions from the tumor to the resection line. This distance is supposed to provide a negative microscopic margin (“no tumor on ink”) and minimal sacrifice of healthy tissue. All published studies on this topic have unequivocally shown that IOUS improves oncological efficacy and cosmetic outcomes in breast conserving surgery [17, 20, 22, 24, 27, 29–31].

The authors present a detailed description of surgical techniques of IOUS-guided breast cancer surgery. Basically, there are two main techniques. The first relies on the continuous use of ultrasound during the operation in order to gauge the distance of the resection line from the edge of the tumor in all directions, without using any ultrasound-visible marker inside or around the tumor. The most detailed description of this technique is given by Krekel et al. [24]. The second technique relies on the use of ultrasound-visible markers as an anatomical landmark [6, 25, 27]. The most detailed description of this technique is given by Ivanovic et al. [27].

However, the majority of resections are still performed using classical palpation-guided surgery, where the desired 10 mm distance from the tumor is subjectively approximated. In practice, one cannot help but notice that most surgeons opt for more extensive resections in order to achieve oncological security. However, a significant percentage of “positive” resection margins makes additional surgery or radiotherapy “boosts” a necessity all too often.

Meanwhile, new studies are being published which confirm that IOUS-guided primary tumor resection is associated with a smaller percentage of positive resection lines. This leads to a reduced need for re-excision and mastectomy, with better esthetic effect and consequently improved quality of life. Volders et al. [30] and Haloua et al. [31] report that this is the consequence of an optimal relationship between volume of the tumor and volume of the excised specimen (tumor and surrounding tissue).

Rubio et al. [32] have shown the advantages of IOUS compared to classical WGL techniques after neoadjuvant chemotherapy. The most important advantages were avoiding the placement of a wire, avoiding the need to synchronize work of diagnostic and surgical teams, allowing for intraoperative confirmation of the specimen (“ex vivo” US examination), and excision of less healthy tissue around lesion with the same margin negativity.

In her article, Klimberg [33] claimed that an excised tumor is rarely in the center of the lumpectomy specimen in daily surgical practice and that ultrasound could help in adequate excision, used in vivo or for specimen examination.

## Conclusions

Over time, the use of IOUS has been transformed from a means of localizing non-palpable lesions to an instrument yielding a reduced number of positive resection margins, with a smaller volume of healthy breast tissue excided around tumor, making the excision of the tumor optimal and objectively measurable.

It seems that intraoperative real-time imaging of breast tumor resection could be the gold standard in the future, after substantial efforts in the education of surgeons and in creating protocols for optimal breast tumor excision.

## Abbreviations

IOUS: Intraoperative ultrasound
WGL: Wire-guided localization
